# Sustained Antiviral and Liver Protection by a Nasal Therapeutic Vaccine (NASVAC, Containing Both HBsAg and HBcAg) in Patients with Chronic Hepatitis B: 2-Year Follow-Up of Phase III Clinical Trial

**DOI:** 10.3390/pathogens10111440

**Published:** 2021-11-05

**Authors:** Sheikh Mohammad Fazle Akbar, Mamun Al Mahtab, Julio Cesar Aguilar, Osamu Yoshida, Eduardo Penton, Guillen Nieto Gerardo, Yoichi Hiasa

**Affiliations:** 1 Department of Gastroenterology and Metabology, Graduate School of Medicine, Ehime University, Ehime 791-0295, Japan; yoshidao.m.ehime@gmail.com (O.Y.); hiasa@m.ehime-u.ac.jp (Y.H.); 2 Department of Hepatology, Bangabandhu Sheikh Mujib Medical University, BSMMU, Dhaka 1000, Bangladesh; shwapnil@agni.com; 3 Center for Genetic Engineering and Biotechnology, Havana 10600, Cuba; julio.aguilar@cigb.edu.cu (J.C.A.); eduardo.penton@cigb.edu.cu (E.P.); gerardo.garcia@cigb.edu.cu (G.N.G.)

**Keywords:** chronic hepatitis B, therapeutic vaccine, sustained effects, nasal vaccine, NASVAC, HBsAg/HBcAg vaccine

## Abstract

A phase III clinical trial in treatment-naïve patients with chronic hepatitis B (CHB) revealed the safety and considerable therapeutic efficacy of a vaccine containing both hepatitis B surface antigen (HBsAg) and hepatitis B core antigen (HBcAg) (NASVAC) at the end of treatment (EOT) and 24 weeks after EOT. Two years after EOT, we checked HBV DNA, alanine aminotransferase (ALT), and hepatitis B e antigen (HBeAg). The data reveal that 33 of 66 NASVAC-recipient CHB patients became negative for HBV DNA in the blood two years after EOT. The ALT levels were within the upper limit of normal (ULN) in 37 patients, although all 66 CHB patients had elevated ALT (above ULN) before the start of therapy. Out of the total twelve HBeAg-positive patients, eight patients became negative for HBeAg. None of the patients developed cirrhosis of the liver within this period. NASVAC is a finite treatment regimen with sustained antiviral and liver-protecting properties. This study is the first to report follow-up data of immune therapy for CHB. NASVAC, an immune therapy of finite duration, is endowed with sustained antiviral and liver protection properties in CHB patients.

## 1. Introduction

Hepatitis B virus (HBV) infection is preventable by vaccination and other public health measures. It is a rational assumption that new cases of HBV will reduce to a satisfactory level within the next few decades [1]. On the other hand, the World Health Organization (WHO) estimates that as of 2019, approximately 296 million people worldwide are currently chronically infected with HBV, expressing HBV DNA and hepatitis B surface antigen (HBsAg) in the blood. Among 296 million chronic HBV-infected subjects, approximately 12–25% exhibit considerable levels of liver damage (elevated alanine aminotransferase (ALT) levels) in addition to harboring HBV DNA and HBsAg, and these patients suffer from chronic hepatitis B (CHB) [2]. CHB patients are prone to developing complications such as cirrhosis of the liver (LC) and hepatocellular carcinoma (HCC) [3]. The estimated number of deaths due to HBV-related liver diseases increased from 786,000 in 2010 to 820,000 in 2019, even after developing a series of drugs with antiviral potentialities for treating CHB patients [2,4]. Most of the professional liver organizations of the world, such the American Association for the Study of Liver Diseases (AASLD) [5], the European Association for the Study of the Liver (EASL) [6], the Asia-Pacific Association for the Study of the Liver (APSL) [7], and other regional and national liver organizations, have recommended two types of drug therapy for treating CHB patients: interferon (standard and pegylated (Peg-IFN)) and nucleos(t)ide analogs (NUCs).

In most cases, NUCs can reduce HBV replication. The use of NUCs has also led to the normalization of ALT in some cases. Investigators have also reported that NUCs can reduce progression to LC and HCC in some patients [8,9]. However, there are several eminent limitations to NUCs. These drugs require long-term usage, or even lifelong in some patients. Discontinuation of NUC treatment induces a flare of HBV DNA and severe hepatitis, which may be life threatening [10].

Considering the severity of HBV-related diseases, the limitation of the efficacy of commercially available antiviral drugs, and the long-term nature of NUC therapy, there is a pressing need to develop novel therapeutic approaches for treating CHB patients. New therapeutic approaches should be evidence based, safe, effective, and endowed with a finite therapeutic option. HBV is a non-cytopathic virus, and liver damage and complications such as LC and HCC are immune mediated; several investigators, including ourselves, have opted to develop immune therapy for CHB with immune modulators since the early 1980s [11]. However, neither polyclonal immune modulators nor HBsAg-based therapeutic vaccines could stand the test of time [12].

After analyzing the design of different immune therapy regimens, we developed a new immune-modulatory drug by mixing HBsAg and hepatitis B core antigen (HBcAg) (NASVAC, Center for Genetic Engineering and Biotechnology (CIGB), Havana, Cuba), and we systematically developed an immune therapy for CHB patients. First, a preclinical study was carried out in HBV transgenic mice that expressed HBV DNA and HBsAg [13]. The safety and efficacy of NASVAC were evaluated in normal volunteers [14]. Finally, we completed a phase I/II clinical trial with NASVAC in CHB patients and checked the mechanism of action of NASVAC, along with its safety and efficacy [15]. Subsequently, phase III clinical trials were completed with NASVAC in treatment-naïve CHB patients [16]. The phase III clinical trial revealed that NASVAC was a safer and more efficacious drug for reducing HBV DNA and achieving the normalization of ALT in CHB patients compared with pegylated interferon 24 weeks after EOT (described in detail in [16]). We found that other studies have provided similar data about immune therapy in CHB patients, but immune therapy for CHB has not received general acceptance due to the lack of follow-up data. Whether immune therapy is safe over a prolonged duration and remains efficacious after EOT remains to be seen.

Here, we provide data about the long-term effects (follow-up data from 2 years after EOT) of NASVAC in CHB patients with regard to HBV DNA and the kinetics of ALT in patients enrolled in a phase III clinical trial. The present communication is the first study of this nature and will provide insights into a finite, safe, efficient, and patient-friendly therapeutic treatment for CHB; a long-waited scientific advancement in the field of drug development for CHB.

## 2. Results

### 2.1. Parameters of Safety

Two years after EOT, no patient had any subjective complaint of adverse effects related to the usage of NASVAC. The serum bilirubin and creatine levels were also below the ULN; thus, any adverse effect of NASVAC on the liver and kidney was discounted. Assessment by ENT (ear, nose, and throat) specialists, as NASVAC was administered via the nasal route, did not reveal any inflammatory change in the nasal mucosa. Assessment of whole blood did not reveal any abnormality in the total counts of blood cells and differential counts. The levels of hemoglobulin were also within the normal range. We also discussed the development of any adverse events related to NASVAC with the patients by direct questioning. None of them reported the occurrence of any such adverse events. Important parameters of safety such as white cell count, levels of bilirubin, albumin, creatinine and platelet counts are shown in Table 1. The patients had fibrosis levels of 1 and 2 in most cases (64 of 66 patients).

### 2.2. Follow Up of Patients with CHB for 2 Years after EOT

The data of 66 patients with CHB will be provided here. These 66 patients could be followed up adequately for two years after EOT, although 78 CHB patients initially took part in the study to receive NASVAC. Thus, 12 patients could not be appropriately followed up, as they missed the follow-up schedule. However, we contacted these 12 CHB patients via telephone, and they were healthy and devoid of any liver-disease-related complaints.

### 2.3. Sustained Control of HBV DNA by NASVAC in CHB Patients 2 Years after EOT

**Figure 1:**
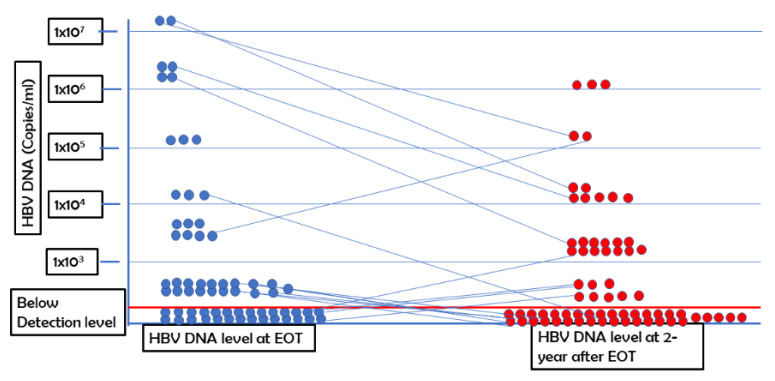
Kinetics of HBV DNA at end of treatment (EOT) and 2 years after EOT.

All patients had at least 1 × 10^3^ copies of HBV DNA in the sera at the start of the phase III clinical trial (16). Out of the 66 patients enrolled this study, HBV DNA was not detectable in the sera of 33 patients (50%) (level of detection; 250 copies/mL) 2 years after EOT. In 30 patients, the levels of HBV DNA in sera 2 years after EOT was reduced compared with the basal levels of HBV DNA. In three patients with CHB, the levels of HBV DNA in the sera increased 2 years after EOT compared with their basal levels. The increased level was 2-log copies in two patients and 3-log in one patient (Figure 1).

The levels of HBV DNA at EOT and 2 years after EOT are shown. Each round dot represents the level of HBV DNA of each patient. The level of HBV DNA is shown on the y axis.

Next, we analyzed the levels of HBV DNA of these patients at 2 years after EOT versus the levels of HBV DNA at EOT. At EOT, the mean level of HBV DNA was reduced significantly compared with the basal levels of HBV DNA; this trend was also described in detail in a previous article (16). The level of HBV DNA at 2 years after EOT was either negative or reduced in 47 patients when compared with those at EOT (Figure 1). We did not find any association between HBV genotype and negativity of HBV DNA at 2-year follow-up.

### 2.4. Kinetics of ALT in Patients Receiving NASVAC

**Figure 2:**
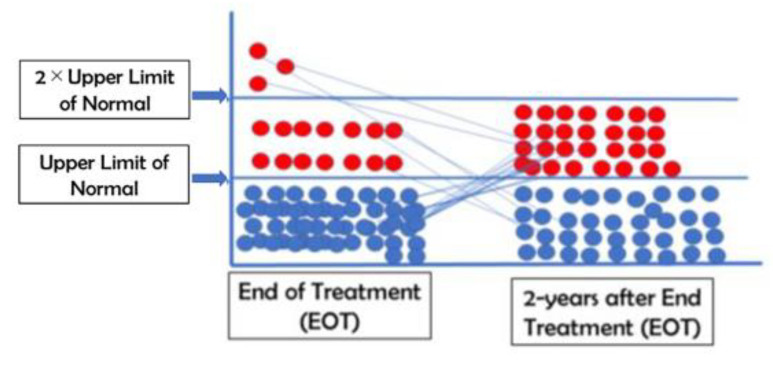
The levels of alanine aminotransferase at end of treatment (EOT) and 2 years after EOT.

As mentioned in a previous article [16], all patients had elevated levels of ALT when they were first enrolled in the study. The ALT initially exhibited unexpected kinetics in these patients. ALT levels increased to twice the upper limit of normal (ULN; 42IU/L)) in 85% of patients receiving NASVAC after five nasal vaccinations. This was potentially related to the induction of immune restoration. Out of a total of 66 patients, the level of ALT was within the upper limit of normal (UNL) in 49 patients. However, the level of ALT was very high in three patients, who possessed a level more than two times higher than the ULN. When the ALT levels of these patients were analyzed two years after EOT, it was found that the levels of ALT were below ULN in 37 patients with CHB. The remaining 29 patients had ALT levels above ULN and below 2 × ULN (43–84 IU/L) (Figure 2). None of the patients had an ALT level more than two times higher than the ULN (Figure 2).

The levels of alanine aminotransferase (ALT) at the end of treatment (EOT) and 2 years after EOT were tabulated.

### 2.5. Role of NASVAC in HBeAg Positivity

There were 12 HBeAg-positive patients within the 66 patients in this cohort. Of these 12, eight CHB patients became negative for HBeAg two years after EOT. This finding is highly encouraging, as no patient became negative to HBeAg at EOT and only four patients were negative for HBeAg when checked 24 weeks after EOT. The data at 2 years after EOT reveal that eight patients were negative for HBeAg at this time. Moreover, five patients developed anti-HBe in their sera. All patients were positive for anti-HBc 2 years after EOT.

### 2.6. Anti-Fibrotic Effects of NASVAC

None of the patients developed cirrhosis of the liver two years after EOT. This was confirmed by a fibro scan and ultrasonography of the upper abdomen. 

The compiled data regarding HBV DNA, ALT, and HBeAg are shown in Table 2.

## 3. Discussion

In 2015, the WHO declared a target of the “Elimination of Hepatitis by 2030” after careful consideration of the present reality of the hepatitis situation worldwide. Regarding the elimination of HBV, the target stipulated that new HBV infection can be reduced by vaccination, harm reduction, and public health measures by 2030 (1). However, significant concerns remain about the treatment of chronic hepatitis B. WHO estimates that 12–25% of the total chronic HBV-infected patients require immediate therapy to contain the development of progressive liver diseases such as LC and HCC (2). Thus, approximately 36–60 million CHB patients require immediate assessment and treatment. However, at present, approximately 2–3 million CHB patients (<1%) have been receiving treatment, and these CHB patients are mostly concentrated in developed and advanced countries (2); several reasons can be given for this. Most of the CHB patients in developing and resource-constrained countries are not aware of their HBV statuses. Next, the currently available antiviral drugs possess significant limitations. These drugs can reduce HBV DNA but are unable to contain complications such as LC and HCC in most cases [17]. In addition, (1) the currently available drugs must be used for a prolonged time or even for life, and (2) the stoppage of the drugs induces hepatic flare, severe hepatitis, and quick progression to LC [18,19]. The situation is highly patient unfriendly for CHB patients in developing countries in Asia and Africa, while more than 80% of CHB patients reside in these countries. 

Thus, there remains a pressing need to develop new and innovative therapy for CHB, and there is a consensus that immune therapy may be an option to treat CHB, either alone or combined with antiviral drugs. The fundamental basis of immune therapy is rationalized; however, concerns remain about designing immune therapy. 

The study presented here has shown that NASVAC, a therapeutic vaccine, is able to induce a sustained reduction in HBV DNA and control ALT in most patients. It also induces HBeAg negativity in approximately 66% of patients and contains progression to LC. The study is endowed with several notable features. NASVAC is a finite therapy and the therapeutic regimen takes only five months. NASVAC can be administered via the nasal route and is patient friendly.

Most importantly, the present report is the first in which follow-up data are provided for an immune therapy. The therapeutic vaccine containing HBsAg was first used in 1994 in CHB patients [20]. In the meantime, different formulations of HBsAg-based vaccines, such as the anti-HBs/HBsAg vaccine complex [21] or cell-based vaccines [22], were used in CHB patients. Some of these studies reported encouraging effects, but none of the investigators provided follow-up data and the question remains as to whether the effect of vaccine therapy is short lived or sustained. Here, we report for the first time on long-term follow-up data of NASVAC. 

The vaccine was shown to be safe for two years after EOT without any adverse effects on the ear, nose, or throat (as it was given by the nasal route), as confirmed by ear, nose, and throat (ENT) specialists. Additionally, the vaccine was safe regarding the general parameters of patients, including liver and kidney functions. It was also highly effective, as demonstrated by the vaccine-induced HBV DNA negativity in more than 50% of patients. The levels of ALT were reduced in most patients. The capacity of the drug was noteworthy in the context of HBeAg negativity, as 66% HBeAg negativity was recorded. Most importantly, the extent of liver fibrosis did not progress, and no patient developed LC.

The first clinical trial with NASVAC was accomplished in Bangladesh in treatment-naïve patients with CHB, with the aim to treat millions of such patients in developing countries. In the meantime, the safety and efficacy of NASVAC were demonstrated in Cuba. An ongoing trial in Japan of NASVAC in NUC-experienced patients has also revealed its safety and clinical efficacy in CHB patients [23]. 

One limitation of the study is the lack of estimation of HBsAg in this clinical trial. The clinical trial was accomplished in Bangladesh, a resource-constrained and developing country with a poor health care delivery system. When the study began, there was no means to assess quantitative HBsAg in Bangladesh; we therefore opted to check quantitative HBV DNA and ALT. Now the trial in Japan has shown that NASVAC reduced HBsAg and induced anti-HBs in a considerable number of CHB patients, indicating that NASVAC can induce the functional care of CHB patients. 

Another important implication of this study is related to its expanded usage in other chronic infections and cancers. Combination therapy with antiviral compounds and immune modulators has been proposed to be a future therapeutic option for containing malignancies [24]. The immune-modulatory capacity of NASVAC and its ability to activate antigen-presenting dendritic cells were shown during a phase I clinical trial (15). There remains a possibility for NASVAC to be used for immune modulation in cancers.

## 4. Materials and Methods

### 4.1. Therapeutic Vaccine: NASVAC

NASVAC is the first therapeutic vaccine licensed for the treatment of CHB and is manufactured under good manufacturing practice (GMP) conditions. It contains 100 μg of HBsAg (Pichia pastoris-derived recombinant HBsAg subtype adw2) and 100 μg of HBcAg (purified Escherichia coli-expressed recombinant full-length HBcAg). NASVAC is administered via intranasal (IN) and subcutaneous (SC) routes and induces broad-base immunity, including mucosal immunity in distal tissues [13,14,15,16]. All patients were of Asian origin and of Bangladeshi nationality.

### 4.2. Phase III Clinical Trial with NASVAC in CHB Patients

The phase III clinical trial was described in detail in our previous publication [16], and a brief description of the trial will be provided here for reference. The phase III clinical trial was conducted at Bangabandhu Sheikh Mujib Medical University, Dhaka, Bangladesh, the only medical university in Bangladesh. Part of the study was performed at Farabi Hospital, Dhaka, Bangladesh. The study was carried out in compliance with the Declaration of Helsinki, and the principles of Good Clinical Practice were properly followed. The project identification code is NO. BSMMU/2010/2363. The date of approval was March 1st, 2010. The name of the ethics committee is “Ethical Review Committee”, Bangabandhu Sheikh Mujib Medical University, Dhaka, Bangladesh. The trial was registered at ClinicalTrials.gov NCT01374308. The patients were registered from a pool of CHB patients expressing HBV DNA and HBsAg in sera, with elevated levels of alanine aminotransferase (ALT) above the upper limit of normal (ULN). Abdominal ultrasonography also revealed that they had been suffering from chronic liver disease, but none of the patients had cirrhosis of the liver (LC). The patients were treatment naïve and had not received any antiviral therapy or immune-modulatory drugs before enrollment in this trial. In fact, they did not receive any drug for the containment of HBV infection. The age of the patients ranged from 18 to 65 years, and both sexes were enrolled. The exclusion criteria included inactive HBV carriers with normal ALT. Patients were negative for hepatitis C virus, hepatitis delta, or human immune-deficiency virus. A history of alcohol or drug abuse within one year before entry or the presence of other hepatic diseases of different etiology were also criteria for exclusion.

According to the study design, a total of 80 patients received NASVAC in two cycles. In the first cycle, a volume of 1.0 mL of NASVAC was administered via the intranasal (IN) route five times using a nasal spray at bi-weekly intervals. In the second cycle, NASVAC was administered five times simultaneously through the IN and subcutaneous (SC) routes. Thus, the entire treatment was finished within five months. 

### 4.3. Design of Long-Term Follow-Up of NASVAC-Recipient CHB Patients

Here, we provide data from two years after EOT of CHB patients receiving NASVAC. Although 78 patients were included in the phase III clinical trial, only 66 patients could be adequately followed up for two years after EOT. The patients attended appointments and communicated with the principal investigator once a month for the first three months after EOT. Then, they were followed up once every three months for the next nine months. After one year of follow-up, the patients were followed up twice a year. Two years after EOT, the patients’ total blood counts were checked. Additionally, the occurrence of any adverse event in the ear, nose, and throat (ENT) was checked by an ENT, as the drug was given via the IN route. Patients were checked for liver and kidney functions. The characteristics of the patient analyzed in this communication is given below (Table 3).

### 4.4. Assessment of HBV DNA, ALT, and Hepatitis B e Antigen (HBeAg) and Abdominal Ultrasonography

In order to obtain uniform and reproducible data, assessments of HBV DNA, ALT, and HBeAg two years after EOT were accomplished by exactly the same methods that were employed at different points of the phase III clinical trial. Anti-HBc, and anti-HBe, were measured in all patients using a commercially available methodology at the hospital specified by the institutional review board. Additionally, the same person performed the abdominal ultrasonography; however, he was not informed about the identity of the patients.

## Figures and Tables

**Table 1:** Parameters of safety of patients receiving NASVAC.

Variables	Basal Level	EOT	2-Year after EOT
White Blood (Counts/mm^3^)	9.0 (5.3–10)	9.0 (5.4–11)	9.0 (5.8–10.0)
Bilirubin (mg/dL)	0.63 (0.2–1.26)	0.7 (0.4–1.16)	0.79 (0.56–0.97)
Albumin (gm/dL)	3.7 (3.4–4.2)	4.1 (3.6–4.6)	3.6 (3.2–4.1)
Creatinine (mg/dL)	0.99 (0.46–1.8)	1.0 (0.62–1.42)	0.89 (0.54–1.27)
Hemoglobulin (gm/dL)	13.2 (12.6–14.2)	12.4 (11.3–15.2)	12.5 (10.6–13.2)
Platelets count	230,000 (190,000–270,000)	210,000 (195,000–245,000)	205,000 (195,000–320,000)

Basal level: The day of 1st injection; EOT: End of treatment; 2 years after EOT: 2 years after end of treatment during follow up.

**Table 2:** The effect of various levels of HBV DNA, ALT, and HBeAg on NASVAC-recipient CHB patients 2 years after EOT.

**(A) Kinetics of HBV DNA**			
**Number of Patients**	**HBV DNA-Negative (<250 copies/mL)**	**HBV DNA Reduced from Basal Levels**	**HBV DNA Increased from Basal Levels**
66	33	30	3
**(B) Kinetics of ALT**			
**Number of Patients**	**Alanine Aminotransferase (ALT) below upper Level of Normal (ULN) (0–42 IU/L)**	**Alanine Aminotransferase (ALT) above ULN and** **2** **×** **ULN** **(43–84 IU/L)**	**Alanine Aminotransferase (ALT) above 86 IU/L**
66	37	29	0
**(C) HBeAg Negativity**			
**Number of Patients**	**HBeAg-Negative**		**HBeAg-Positive**
12	8		4

ALT, alanine aminotransferase; HBeAg, hepatitis B e antigen; EOT, end of treatment.

**Table 3:** Patient characteristics on the day prior to administration of 1st dose of NASVAC.

Variables		Values
Total		66
Male: Female		59:7
Age (Years)		20 (18–50)
HBV DNA (copies/mL)		4.33 × 10^3^ (range: 1.5 × 10^3^–1.0 × 10^13^)
ALT (IU/L) *		30 (10–262) *
HBeAg-positive		12

* The value of ALT just prior to administration of 1st dose of NASVAC are shown.

## Data Availability

All data can be available from Prof. Mamun Al Mahtab, Head, Department of Hepatology, Bangabandhu Sheikh Mujib Medical Univesity (BSMMU), Dhaka, Bangladesh (shwapnil.agni.com). He is the principal Investigator of the study.
